# Perspective on donor‐derived infections in Italy

**DOI:** 10.1111/tid.14398

**Published:** 2024-10-15

**Authors:** Paolo Antonio Grossi, Letizia Lombardini, Raffaele Donadio, Daniela Peritore, Giuseppe Feltrin

**Affiliations:** ^1^ Department of Medicine and Surgery Infectious and Tropical Diseases Unit University of Insubria‐ASST‐Sette Laghi Varese Italy; ^2^ Italian National Center for Transplantation Istituto Superiore di Sanità Rome Italy

## Abstract

**Background:**

Expected and unexpected donor‐derived infections (DDI) are a rare event in solid organ transplant (SOT) recipients but are potentially associated with significant morbidity and mortality. To assure the microbial safety of transplantation, both national guidelines and the current, regional, and local epidemiology of infectious diseases must be considered.

**Methods:**

In the present paper the strategies adopted by the Italian National Center for Transplantation (CNT) since 2003 and their evolution to guarantee the safety of organ transplantation are reported. Starting in 2012 mandatory reporting to the CNT of all adverse reactions was started. The number and type of DDI reported to the CNT are currently being analyzed.

**Results:**

The infectious diseases second opinion has written and updated the guidelines on the safety of organs for transplantation and supported the Italian transplant network for the use of organs from donors with suspected or documented potentially transmissible infections.

**Conclusion:**

A transplant safety network was developed in Italy in 2003 and has been updated according to the evolving knowledge and the changing epidemiology. This is an evolving field, and a continuous update of the recommendation is needed.

**List of Abbreviations**
CNT: Italian National Center for Transplantation
COVID‐19: coronavirus disease 2019
DDI: donor‐derived infections
HBV: hepatitis B virus
HBsAg: hepatitis B surface antigen
HCV: hepatitis C virus
HIV: human immunodeficiency virus
IgG: immunoglobulin G
IgM: immunoglobulin M
MDRO: multidrug‐resistant organism
NAT: nucleic acid testing
PCR: polymerase chain reaction
PF: preservation fluid
SARS‐CoV‐2: severe acute respiratory syndrome coronavirus 2
SOT: solid organ transplant
WNV: West Nile Virus

## 1 INTRODUCTION

Solid organ transplant (SOT) recipients are at risk for expected or unexpected donor‐derived infections (DDI), which may be graft‐specific or systemic.^1^ According to the most recent report of the Disease Transmission Advisory Committee in the U.S.A., unexpected DDIs were rare and occurred in 0.18% of all SOT recipients. However, graft loss or death occurred in about one‐third of recipients with DDIs, with higher rates associated with parasitic and fungal diseases.^2^

In 2001, the Italian National Center for Transplantation (CNT), which is responsible for the procurement, processing, and distribution of organs and tissues in Italy, created a national commission of experts in different medical‐surgical specialties according to Italian law 91/99, with the mission of defining guidelines for the evaluation process of the potential organ donor. In September 2003 the text of the guidelines was approved by the CNT as a technical annex of the Ministry Decree of August 2, 2002. In November 2003 the text was also officially approved by the State‐Region conference between the Health Ministry and the Regional Health Departments.^3^ The guidelines have been updated periodically and the last version was released in January 2024.^4^ In addition, together with the first release of the guidelines, the CNT has supported the transplant network through an expert task force (second opinion) for evaluation of marginal cases. The second opinion task force is formed by a panel of professional experts nominated by a Ministerial Decree dated October 27, 2004, confirmed in the subsequent years by Decrees of the Director of the Italian CNT.^5^

Decisions regarding the utilization of organs from a donor with known infection depend on multiple factors including the risk for disease transmission and the availability of therapies should such transmission occur. Organs are commonly used from donors carrying treated infections due to bacteria or fungi or with treatable viral infections including hepatitis B virus (HBV), hepatitis C virus (HCV), or human immunodeficiency virus (HIV). This risk is considered acceptable given the availability of sensitive diagnostic assays and effective prophylactic or pre‐emptive therapies.

### 1.1 Microbial safety

In order to guarantee the safety of organs for transplantation the Italian guidelines require that each donor is screened according to what is reported in Tables 1 and 2. In addition, specific testing for infections is required for donors with certain geographic connections (individuals who have lived in and/or traveled to endemic areas (with a risk of being exposed to pathogens) or are at risk for vertically acquired infection (where appropriate). A review of the available information (e.g., medical, including use of illicit drugs, epidemiological and social history, ethnicity, and travel history) should guide the decision‐making process as to which specific screening tests to deploy beyond the mandatory markers; a balanced approach is required. For donors whose medical history is positive for travel and/or native from countries where the microbial agents listed in Table 3 (modified from the Council of Europe Guide to Quality and Safety of Organs for Transplantation, 8th edition)^6^ are endemic, in the absence of specific indications from the CNT, it is mandatory to contact the national infectious diseases second opinion. However, it is impossible to exclude all risks for unexpected disease transmission.

**TABLE 1:** List of mandatory laboratory tests that are required prior to organ procurement.

Test	Supplementary tests
HIV 1 e 2 (antibodies)	Western blot or HIV‐RNA if positive
HCV (antibodies)	HCV‐RNA if positive
HBV (HBsAg, HBsAb, and HBcAb)	If HBsAg and/or HBcAb positive: HBsAb and HBV‐DNA If HBsAg positive anti‐HDV (antibodies) and if positive HDV‐ RNA.
Syphilis serology or TPHA or TPPA	VDRL or RPR if positive. If both screening test and VDRL or RPR are positive mandatory HIV‐RNA, HBV‐DNA, and HCV‐RNA before procurement.
Bronchoalveolar lavage or sample from the lower respiratory tract for SARS‐CoV‐2‐RNA detection with molecular testing. Performing a nasopharyngeal swab is not required. If it is available, and with a result that is discrepant with that of the BAL, it is necessary to contact the national second opinion.	This test is currently mandatory for all organ donors but might change in the near future for non‐lung donors.
HTLV 1 and 2 antibodies if specific risk factors	If positive confirmatory test by western blot or HTLV‐RNA.
Blood, urine, sputum, or other respiratory sample cultures, rectal swab (even in the absence of clinical signs of infection, on the day of donation. The results, even preliminary, must be promptly transmitted to the Regional Transplant Center and to CNT. Further investigations, culture, serological, or molecular, aimed at the search/exclusion of specific pathogens will be carried out in the light of specific clinical pictures on the indication of the infectious second opinion.	
Additional molecular testing investigations for donors for whom the medical history, physical examination, or results of laboratory tests reveal doubts: HIV‐RNA and/or HCV‐RNA and/or HBV‐DNA HEV‐RNA (on blood and liver tissue only in the case of donors with hepatitis of unknown etiology).	
Search for HSV‐DNA, VZV‐DNA, HHV‐6‐DNA, CMV‐DNA, EBV‐DNA, Enterovirus‐RNA, and West Nile Virus‐RNA (in the summer months) on blood and CSF for donors with neurological symptoms and suspected diagnosis of encephalitis.	

Abbreviations: BAL, Bronchoalveolar lavage; HCV, Hepatitis C virus; HBV, Hepatitis B virus; HDV, Hepatitis D virus; HIV, Human immunodeficiency virus; TPHA, Treponema pallidum hemo agglutination; TPPA, Treponema pallidum particle agglutination; RPR, rapid plasmin reagin; VDRL, Venereal Disease Research Laboratory.

**TABLE 2:** List of mandatory laboratory tests that may be acquired after organ procurement.

Test	Supplementary tests
Anti‐CMV IgG	In the absence of a suspicion of an ongoing infection, determination of IgM antibodies is not required. In the event that it is carried out and with a positive result, it is necessary to determine the corresponding viremia, through molecular testing.
Anti‐HSV‐1 and 2 IgG	
EBV (VCA‐IgG and EBNA)	
Anti‐VZV IgG	
Anti‐Toxoplasma IgG	
*Strongyloides stercoralis* serology	
Search for ureaplasma and mycoplasma by molecular testing or culture on BAL or deep respiratory secretions for lung donors (the results of these tests must be acquired as soon as possible after transplant). The sample for testing must be taken from the donor by the procurement team.	

Abbreviations: CMV, Cytomegalovirus; EBV, Epstein Barr Virus; HSV, Herpes simplex virus; VZV, Varicella zoster virus.

**TABLE 3:** Additional tests that should be considered for donors with certain geographic connections (modified from 6). The tests listed should be considered for screening donors who are symptomatic and who have lived in and/or traveled to endemic areas (with risk of being exposed to pathogen) or are at risk of vertically acquired infection (where appropriate). When required, these tests are usually done soon after transplantation, so follow local donor screening protocols; whenever appropriate, seek advice from a transplant infectious disease expert at early stages during donor characterization. Refer also to the section on the specific pathogen.

Pathogen	Central and South America	North America	North Africa	Sub‐Saharan Africa	Indian subcontinent	Southeast Asia	Europe	Parameter tested^‡^
*HTLV*	Always		Always	Always	Always	Always	Romania	HTLV‐1/2 antibodies if reactive, confirmation by western blot or NAT
Stool examination for parasites	Always		Always	Always	Always	Always		Stool examination in deceased donors of limited practicality
Urine examination for parasites			Egypt	Always				
*Plasmodium* spp.	Central America and Amazon			Always	Always	Always		DNA (limited availability for screening); for previous exposure: serology for antibodies (with or without NAT)
*Strongyloides stercoralis*	Always	Appalachian and Southern States	Always	Always	Always	Always	(Southern Europe)	Serology
*Schistosoma* spp.	Caribbean, Venezuela, and Brazil		Always	Always		Always		Serology
*Trypanosoma cruzi*	Always (not Caribbean)							NAT or Strout test for exclusion of parasitemia, serology for screening
*Leishmania*	Always		Always	Always	Always	Always		NAT and serology
*Paracoccidioides brasiliensis*	Brazil							If additional data raise concerns of infection: serology
*Blastomyces* spp.		Eastern USA/Canada						
*Coccidioides* spp.	Always	USA western desert and northern Mexico						If resident: Serology (urine antigen if available)
*Histoplasma capsulatum*	Always	Ohio/Mississippi River valleys		Always	Always	Always		If additional data raise concerns about infection: serology (urine antigen if available)
*Babesia microti* and *Anaplasma Borrelia burgdorferi*		New England						Refer to tests for human granulocytic *anaplasmosis* and *Lyme borreliosis*

Abbreviation: HTLV, Human T‐cell leukaemia/lymphoma virus type 1 and type 2.

### 1.2 Infectious diseases second opinion

Since the first release of the Italian quality and safety guidelines, the CNT has supported the transplant network through an expert task force (second opinion) for the evaluation of doubtful cases. The second opinion task force is formed by a panel of professional experts in infectious diseases, pathology, hematology, critical care medicine, forensic medicine, clinical immunology, and rare diseases. The experts are available 24/7 and provide support to the National Transplant Network, and it is the consulting tool used by the local coordinator during the evaluation of the donor or single organ. In addition, they act as a reference point for transplant surgeons and physicians in the management and monitoring of recipients from donors with potentially transmissible diseases. As an example, the infectious disease second opinion is requested for 32% of all potential organ donors with a mean of 73 consultations per month.^7^ In addition to the support to the Italian transplant network is their responsibility to periodically update the national guidelines.

### 1.3 Optimization of antimicrobial perioperative prophylaxis in SOT

Perioperative prophylaxis during organ transplantation is used mainly to prevent surgical site infections as in non‐transplant surgeries, but it may be useful to prevent DDI as well.^8^ If a donor has a documented infection like bacteremia, meningitis, endocarditis, or other potentially transmissible infections the second opinion provides suggestions on how to modify the perioperative prophylaxis in order to prevent transmission. In general, the modified perioperative prophylaxis is continued until the results of the cultures collected at the time of organ procurement are available. If negative the prophylaxis is stopped while if positive the prophylaxis is switched to early treatment and modified according to the sensitivity of the isolated pathogen.

### 1.4 Management of positive cultures of preservation fluid

Preservation fluid (PF) cultures are not routinely performed and the modality of preservation fluid collection is not standardized. The role of PF cultures in predicting post‐transplant infections and the relative mortality in transplant recipients is debated.^9^ In a recent meta‐analysis, 13% of PF was found to be contaminated by a pathogenic organism (95% confidence interval 9.0%–17.0%), with a low incidence of PF‐related infections (4%), but high mortality (35%) leading the authors to recommend routine PF cultures during procurement and transplantation.^10^ Isolation of potential contaminants such as coagulase‐negative Staphylococci is generally ignored, however, isolation of multidrug‐resistant organisms (MDROs) may be associated with transmission despite negative donor cultures.^11^ Although not standardized, we recommend PF cultures whenever possible with a careful interpretation of the results made by a transplant infectious disease specialist, particularly if an MDRO is isolated.

### 1.5 West Nile Virus

SOT recipients are at higher risk of developing neuroinvasive disease with West Nile virus (WNV) infections and can acquire infection via organ transplantation. Several reviews of donor‐derived WNV infection report a high incidence: 87% of the recipients of an organ from a positive donor were diagnosed with infection; 75% of the infected recipients had neuroinvasive disease.^12^ For the first time in Italy, two patients with meningoencephalitis were diagnosed with WNV infection in September 2008.^13^ The patients lived in the Bologna and Ferrara provinces of Emilia Romagna where WNV infections had previously been noted in horses, crows, and magpies.^14^ Since 2008 West Nile virus has become endemic in Italy and the first donor‐to‐recipient transmission occurred in 2009.^15^ The infection was diagnosed in the liver recipient on day 3 after transplantation due to the mandatory screening for WNV released by the CNT for all organ donors^16^ the day after the liver transplant was performed. Since 2008, from June to November all organ donors living in areas where WNV had been demonstrated to be endemic have to be screened by nucleic acid testing (NAT) within 72 h of organ donation. More recently the start of the screening has been related to the report of the first human case in the area. In 2011 a second WNV transmission to four recipients occurred in north‐eastern Italy from a multiorgan donor who was negative by NAT but immunoglobulin G (IgG) and immunoglobulin M (IgM) were positive.^17^ Due to the low level and transient viremia during WNV infection NAT should be enhanced by the search for IgG and IgM antibodies. However, these tests can be carried out in a limited number of reference laboratories, to ensure high quality standards, and it is therefore impossible to obtain the results for every organ donor. Fortunately, since 2011 no further case of WNV transmission has been reported in Italy.

### 1.6 HBV infection

In all previous editions of the Italian guidelines, hepatitis B surface antigen (HBsAg)‐positive donors could donate only to HBsAg‐positive recipients or to HBV naïve recipients only for life‐saving procedures. In recent years, we have allowed heart, lung, and liver transplantation from HBsAg‐positive deceased donors and kidney transplantation from HBsAg‐positive living donors to vaccinated or naïve recipients with prophylaxis with anti‐HBV immunoglobulin and antiviral treatment with no transmission (manuscript in preparation). In the last edition of the Italian guidelines, released in January 2024, transplantation of any organ from HBsAg positive donor is now allowed to any recipient, regardless of the HBV immune status, provided that a combined prophylaxis is administered to the recipients.^4^

### 1.7 HIV‐positive donor

In recent decades, antiretroviral therapy (ART) has dramatically changed the survival of the population living with HIV infection and currently, many HIV subjects have a life expectancy similar to that of the general population. The increase in survival has led to a significant increase in chronic diseases that in this patient population are more frequent and rapidly evolving. Numerous studies show, in patients with HIV/HCV co‐infection, a rate of progression to cirrhosis that on average is much higher than compared to non‐co‐infected subjects, resulting in end‐stage liver failure which is the most common cause of death among HIV‐infected patients. Chronic kidney disease is also very common in these subjects with an increase in patients developing renal insufficiency and therefore needing to resort to dialysis treatment. Although less frequent, terminal heart and lung diseases, which can only be treated with a transplant, are a nosological entity in constant growth. Since data on SOT from donors HIV positive to HIV‐infected recipients looked promising,^18^, ^19^, ^20^, ^21^ in 2017 we started using kidneys and livers for HIV‐infected individuals with special permission from the Ministry of Health and in 2018 it was issued by the Ministry of Health, the DECREE of February 1, 2018 “Amendment of Article 3 of the Decree of August 2, 2002, entitled: ‘Criteria and procedures for certifying the suitability of organs harvested for transplantation (Article 14^5^ of Law No 91 of April 1, 1999)’. (18A01639) (OJ General Series No. 56 of 08‐03‐03) 2018)” which also authorized this activity in Italy, the first European country to adopt specific criteria for the eligibility of the potential HIV‐positive donor, reported below:

1. Donor followed by an infectious disease facility.
2. If the donor is receiving antiretroviral therapy, documented efficacy of the therapy (undetectable HIV‐RNA).
3. Absence of opportunistic and neoplastic pathologies.
4. If possible, the suitability of the organ is documented by histological findings.
5. No a priori restrictions for CD4+ lymphocyte counts.
6. Possibility for the infectious disease team to identify an appropriate treatment regimen antiretroviral (cART) to be initiated in the recipient, based on the clinical and pharmacological history of the donor and the recipient.

The donor with HIV infection, who meets the criteria listed above, is considered at standard risk for HIV‐infected recipients unless other conditions coexist.

At the end of 2023, a total of 15 transplants from HIV‐positive donors were performed in Italy (nine livers and six kidneys) with outcomes comparable to HIV‐positive recipients of organs from HIV‐negative donors (unpublished data).

### 1.8 Strongyloides stercoralis

*Strongyloides stercoralis* is estimated to infect over 300 million people globally.^22^ Transmission of Strongyloides from the donor has been reported after heart, kidney, kidney‐pancreas, and liver transplantation.^23^, ^24^ The fatality rate of hyperinfestation syndrome exceeds 50%. A recent epidemiological study conducted in Italy showed positivity rates of serology of 8% in the Italian population and 17% in the foreign population.^25^ In the last edition of the Italian guidelines it is therefore recommended to carry out serology (IgG antibodies) for *S. stercoralis* on all organ donors, the result of which is not necessary for organ allocation but may allow early treatment of the recipients. The test result must be available, if possible, within 72 h of transplantation. From January to April 2024, we have already identified eight donors with positive serology and all recipients have been treated with prophylactic Ivermectin with no transmission.

### 1.9 Strategies during the coronavirus disease 2019 pandemic

Since the beginning of the coronavirus disease 2019 (COVID‐19) pandemic, the CNT has released recommendations on donor testing recommending deceased donor screening by polymerase chain reaction (PCR) on lower respiratory tract infection regardless of the procured organs. This policy is still active, although we are considering limiting the screening only to lung donors. Starting from May 2020 we started using organs from donors with resolved COVID‐19 (at least 14 days after the first negative PCR on a nasopharyngeal swab; starting from January 2022 we have reduced the interval to 7 days). In November 2020 we started using hearts and livers from donors with active severe acute respiratory syndrome coronavirus 2 (SARS‐CoV‐2) infection for recipients with resolved SARS‐CoV‐2 infection, assuming a protective immunity. In fact, no transmission was observed in the recipients.^26^, ^27^ In January 2022, we have extended the use of hearts, livers, and kidneys to recipients either with past COVID‐19 or COVID‐19 vaccination with at least three doses of an mRNA vaccine. Again, no transmission was observed in all the recipients. It is interesting to underline that in a small series of liver transplant recipients from donors with active SARS‐CoV‐2 infection, at multivariate analysis COVID‐19 donor was the only factor significantly and independently associated with hepatic artery thrombosis.^28^ This observation needs to be confirmed in larger multicenter studies.

### 1.10 Analysis of adverse events and adverse reactions in the experience of the Italian National Transplant Network

An adverse event is an undesired and unexpected occurrence associated with any stage of the chain from donation to transplantation that might lead to harm in SOT recipients or living organ donors while an adverse reaction is an unintended response, including a communicable disease, in the recipient or in the living donor that might be associated with any stage of the chain from donation to transplantation.

The CNT has been collecting reports of adverse events and reactions since 2012. We are currently analyzing these data that we believe are underestimated, particularly regarding the transmission of infections. Unfortunately, the analysis is still ongoing due to the lack of follow‐up data and therefore we are unable at the moment to share the results that will be published when all the follow‐up of the recipients will be collected and the analysis will be completed.

## 2 CONCLUSION

SOT recipients are at risk for expected and unexpected DDI. A safety system is mandatory to guarantee successful transplantation without arms to the recipients. The Italian transplant network has developed a very efficient system, with the support of the second opinion, that allows to use of organs from donors with potentially transmissible infections without transmission to the recipients. The only adverse reactions observed were unexpected and related to the use of organs procured from standard risk donors. The impact on graft and patient survival of these events is currently under evaluation.

## AUTHOR CONTRIBUTIONS

Paolo Antonio Grossi wrote the manuscript; Letizia Lombardini, Raffaele Donadio, Daniela Peritore, and Giuseppe Feltrin revised the manuscript.

## CONFLICT OF INTEREST STATEMENT

The authors declare no conflict of interest.

## FUNDING INFORMATION

The authors received no financial support to produce this manuscript.

## Data Availability

Data sharing is not applicable to this article as no new data were created or analyzed in this study.
